# Supplementary material for: CoA synthase regulates mitotic fidelity via CBP-mediated acetylation
Source: Nat Commun. 2018 Mar 12;9:1039. doi: 10.1038/s41467-018-03422-6 (PMC5847545; doi:10.1038/s41467-018-03422-6)
Supplement: Supplementary file 1 — Supplementary Information [file 41467_2018_3422_MOESM1_ESM.pdf]

Supplementary Information

CoA Synthase Regulates Mitotic Fidelity via CBP-Mediated Acetylation

Lin et al.

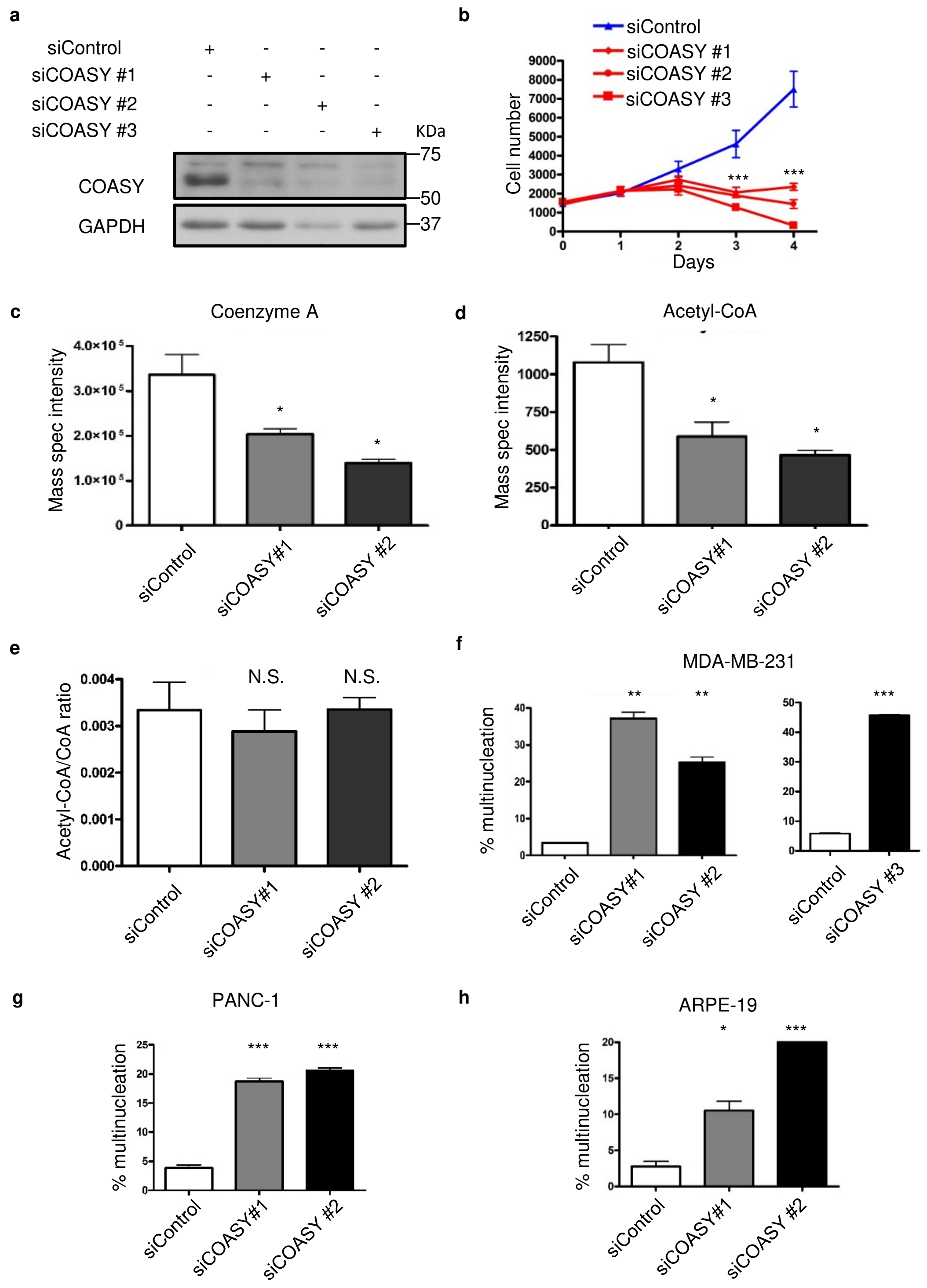

Supplementary Fig. 1 COASY knockdown decreased cell numbers, CoA, acetyl-CoA and increased multi-nucleation. **a** COASY siRNAs reduced the levels of COASY protein. MDA-MB-231 cells were transfected with COASY siRNAs for 3 days before being lysed and analyzed by Western blots. **b** COASY knockdown reduced cell numbers of MDA-MB-231 cells. Indicated siRNAs were transfected to MDA-MB-231 cells (1500 cells/well) and the cell numbers were determined at indicated days. Two-way ANOVA: $p<0.0001$. Bonferroni post hoc tests, ***$p<0.001$. n=3 independent repeats. **c-e** COASY knockdown reduced the levels of CoA and acetyl-CoA. COASY knockdown did not alter the Acetyl-CoA/CoA ratio. The CoA and acetyl-CoA level of A549 cells as quantified by mass spectrometry. **f-h** The percentage of MDA-MB-231 (**f**) PANC-1 (**g**) and ARPE-19 (**h**) that exhibit multi-nucleation phenotypes 72 hours after transfection with control or two independent COASY siRNAs. For each sample, more than 150 cells were scored by immunofluorescence microscopy. *$p<0.05$, **$p<0.01$, ***$p<0.001$, two-tailed Student's t-test, n=3 independent repeats. Bars show standard error of the mean.

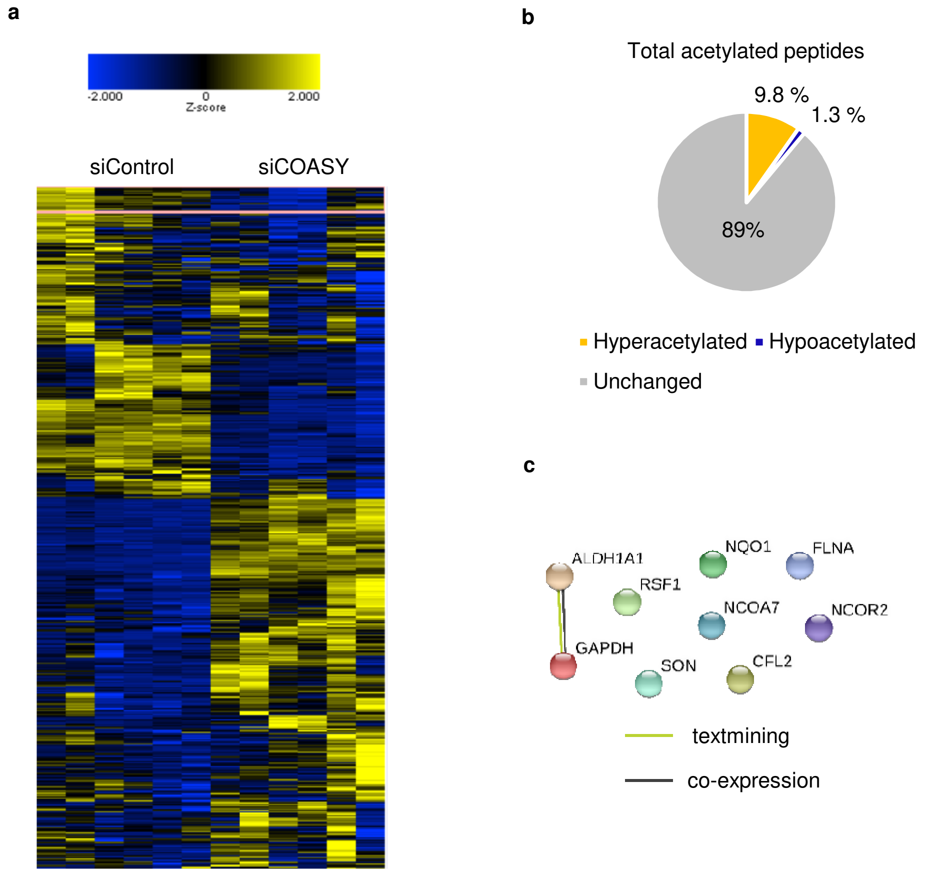

Supplementary Fig. 2 COASY knockdown leads to hyper-acetylation of a protein network. **a** Heatmap shows all the acetylated peptides (n=1074) across all samples transfected with siRNAs targeting Control or COASY. The color (yellow: induced, blue: repressed) indicates the normalized Zscore in Supplementary Table 1. **b** Pie chart shows the percentage of hyperacetylated and hopoacetylated peptides by using a cut-off value of p<0.001 in two-tail Students t-test. **c** Hypoacetylated proteins under COASY knockdown generated using STRING database.

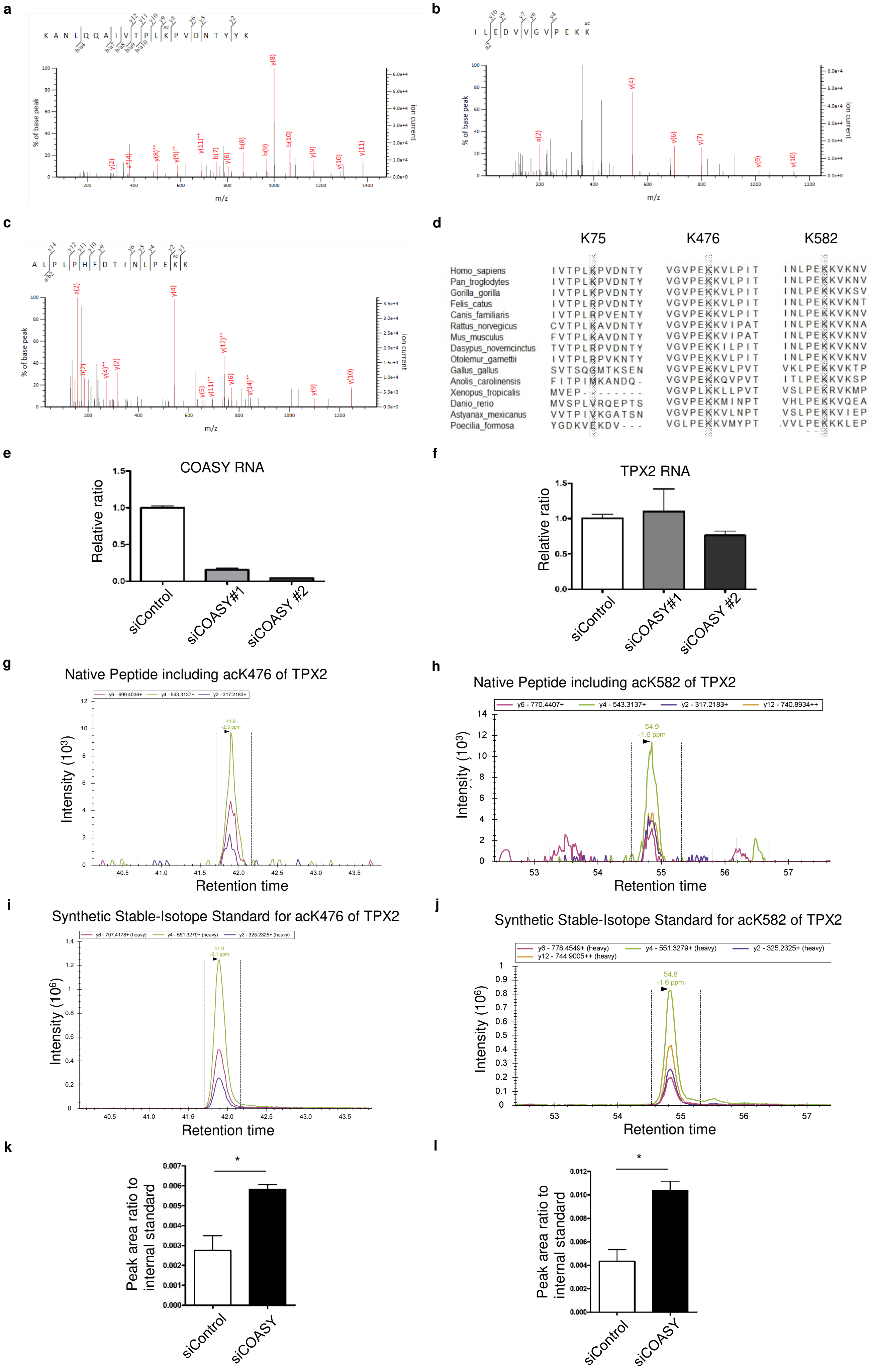

a
KANLQQAIVTPLKPVDNTYK
% of base peak
Ion current
m/z
b
ILEDVVGVPEKK
% of base peak
Ion current
m/z
c
ALPLPHFDTINLPEKK
% of base peak
Ion current
m/z
d
K75
K476
K582
Homo_sapiens IVTPLKPVDNTY VGVPEKKVLPIT INLPEKKVKNV
Pan_troglodytes IVTPLKPVDNTY VGVPEKKVLPIT INLPEKKVKNV
Gorilla_gorilla IVTPLKPVDNTY VGVPEKKVLPIT INLPEKKVKSV
Felis_catus IVTPLRPVDNTY VGVPEKKVLPIT INLPEKKVKNT
Canis_familiaris IVTPLRPVENTY VGVPEKKVLPIT INLPEKKVKNV
Rattus_norvegicus CVTPLKAVDNTY VGVPEKKVIPAT INLPEKKVKNA
Mus_musculus FVTPLKAVDNTY VGVPEKKVIPAT INLPEKKVKNV
Dasypus_novemcinctus TVTPLRPVDNTY VGVPEKKILPIT INLPEKKVKNV
Otolemur_garnettii IVTPLRPVKNTY VGVPEKKVLPIT INLPEKKVKNV
Gallus_gallus SVTSQGMTKSEN VGVPEKKVLPVT VKLPEKKVKTP
Anolis_carolinensis FITPIMKANDQ- VGVPEKKQVPVT ITLPEKKVKSP
Xenopus_tropicalis MVEP-------- VGVPLKKLLPVT VSLPEKRVKMP
Danio_rerio MVSPLVRQEPTS VGVPEKKMINPT VHLPEKKVQEA
Astyanax_mexicanus VVTPIVKGATSN VGVPEKKVLNPT VSLPEKKVIEP
Poecilia_formosa YGDKVEKDV--- VGLPEKKVMYPT VVLPEKKKLEP
e
COASY RNA
Relative ratio
siControl
siCOASY#1
siCOASY #2
f
TPX2 RNA
Relative ratio
siControl
siCOASY#1
siCOASY #2
g
Native Peptide including acK476 of TPX2
Intensity (10^3)
Retention time
h
Native Peptide including acK582 of TPX2
y6 - 770.4407+
y4 - 543.3137+
y2 - 317.2183+
y12 - 740.8934++
Intensity (10^3)
Retention time
i
Synthetic Stable-Isotope Standard for acK476 of TPX2
Intensity (10^6)
Retention time
j
Synthetic Stable-Isotope Standard for acK582 of TPX2
y6 - 778.4549+ (heavy)
y4 - 551.3279+ (heavy)
y2 - 325.2325+ (heavy)
y12 - 744.9005++ (heavy)
Intensity (10^6)
Retention time
k
Peak area ratio to internal standard
siControl
siCOASY
l
Peak area ratio to internal standard
siControl
siCOASY

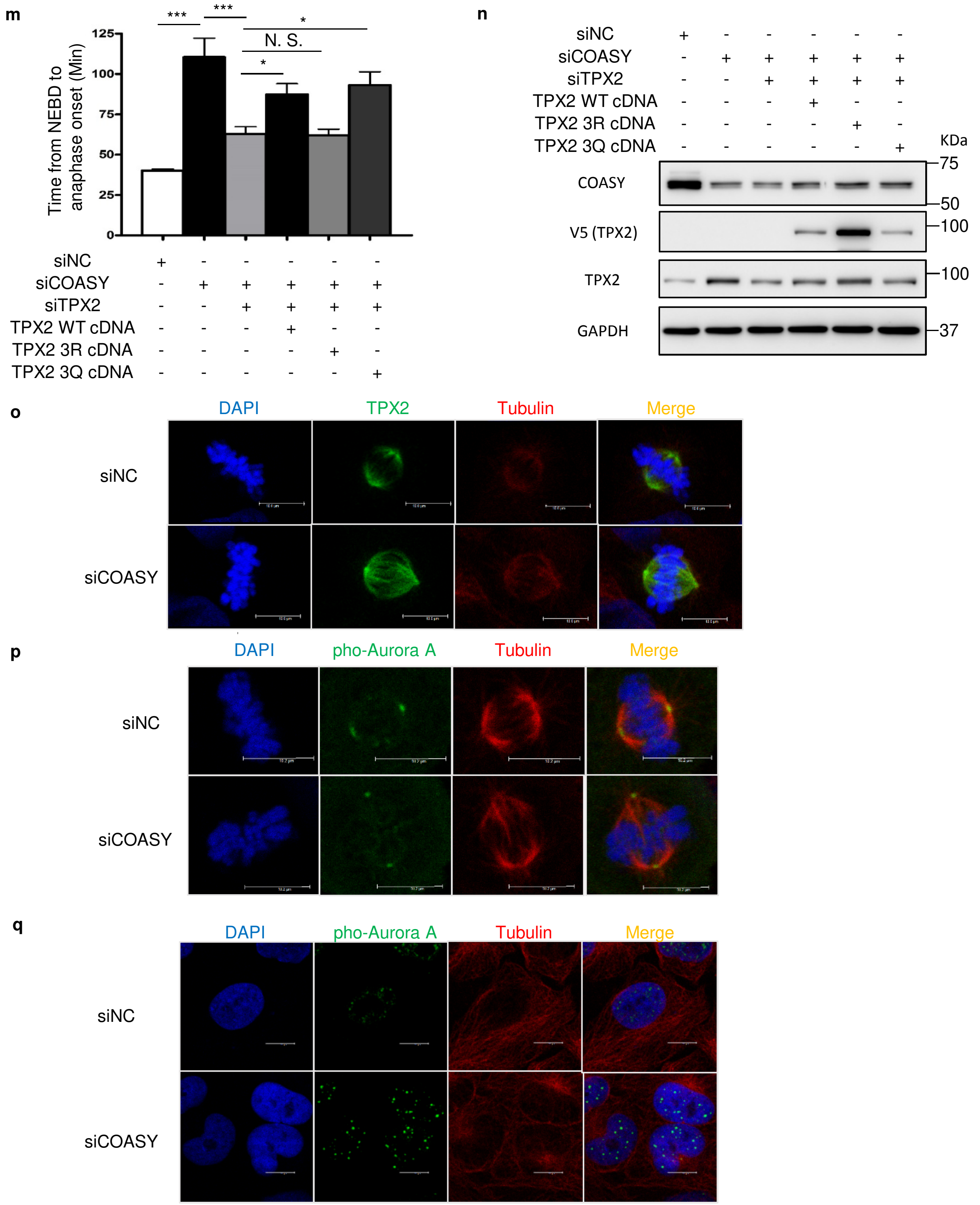

Supplementary Fig. 3 a-c Acetylome analysis identified three potential acetylation residues on TPX2. MS/MS Spectra Images of TPX2 K75 (**a**) K476 (**b**) and K582 (**c**). **d** Two of the three potential acetylation sites of TPX2 are highly conserved among 15 different examined species. **e, f** COASY knockdown reduced COASY RNA (**e**), but did not alter TPX2 RNA expression (**f**). A549 cells transfected with siControl or siCOASY were enriched in mitosis by thymidine-nocodazole block. The levels of COASY and TPX2 transcripts were then determined by Real-Time PCR. **g, h** PRM chromatogram of the native acetylated TPX2 peptides including K476 (**g**) and K582 (**h**). **i, j** PRM Chromatogram of the stable-isotope internal standard acetylated TPX2 peptides including K476 (**i**) and K582 (**j**). **k, l** Quantification of acetylation of K476 (**k**) and K582 (**l**) on TPX2 using PRM. COASY knockdown increased the acetylation of both K476 and K582. Quantification of the native acetylated TPX2 peptides , calculated as a ratio to the stable-isotope internal standard peptide spiked prior to sample digestion and IP, was performed using PRM targeted mass spectrometry. n=3 for each group. **m** The acetylated lysines on TPX2 regulated the percentage of COASY-dependent extended mitosis. TPX2 knockdown rescued the extended mitosis induced by COASY knockdown. Reintroducing wild type (WT), or acetylation-mimetic (3Q), but not acetylation-deficient (3R), mutant of siRNA-resistant TPX2 cDNA recapitulated extended mitosis phenotype. **n** Western blot for validating the expression of indicated proteins in Fig 3G and Supplemental Fig S3G. **o** During metaphase in A549 cells, TPX2 had high intensity in spindle poles under confocal microscopy. Upon COASY knockdown, more TPX2 signal was observed extending from spindle poles to chromosomes. **p** COASY knockdown did not alter Aurora A phosphorylation during metaphase. **q** COASY knockdown led to stronger Aurora A phosphorylation was observed in the nuclei during interphase. Scale bars, 10 µm. (**k-m**) $*p<0.05$, $***p<0.001$, two-tailed Student's t-test, (**k, l**) n=3 independent repeats. (**m**) Representative figure of 2 experiments. n=100 cells were monitored for each group, Bars show standard error of the mean.

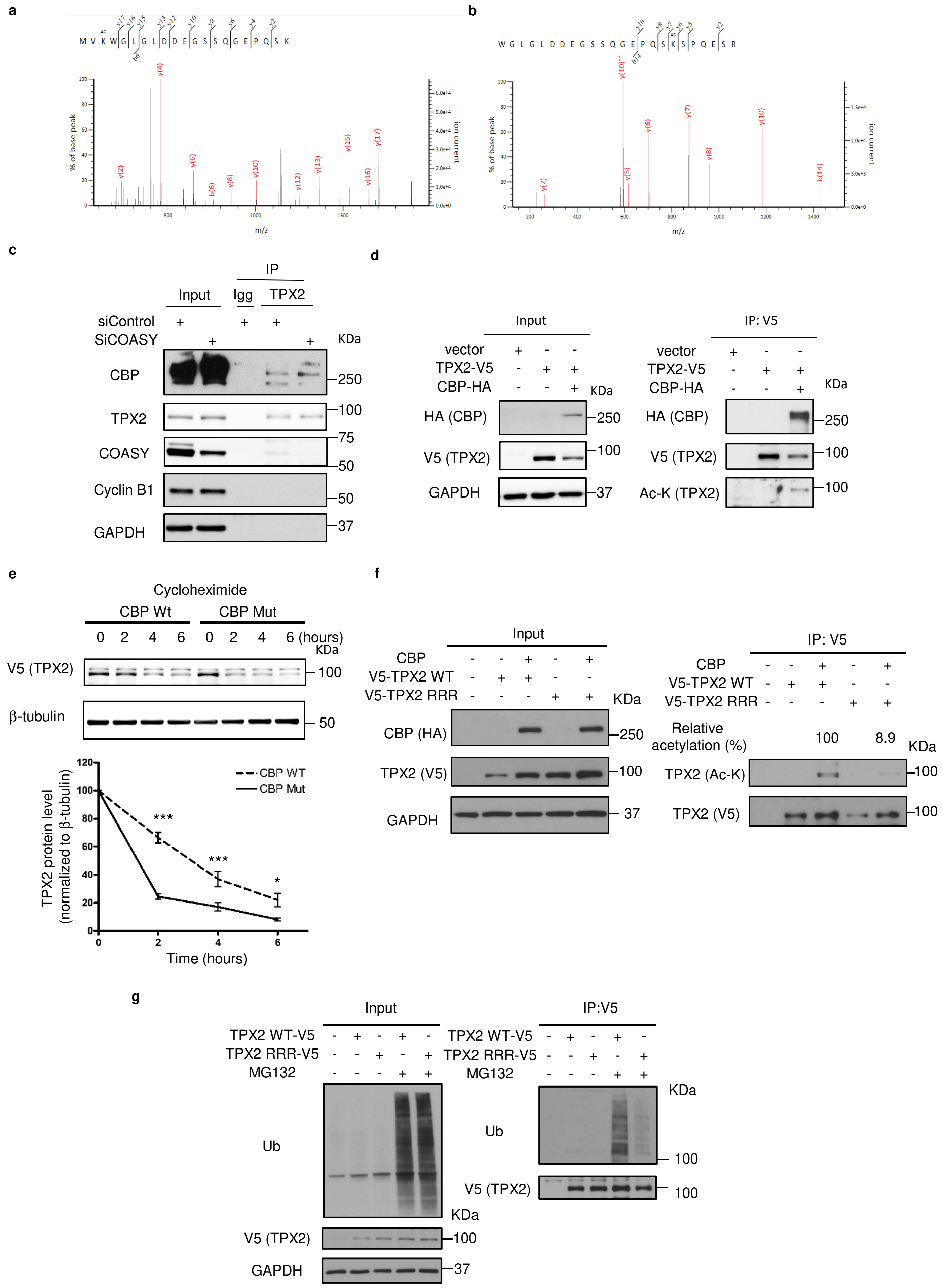
a
M V K W G L G L D D E G S S Q G E P Q S K
% of base peak
ion current
m/z
b
W G L G L D D E G S S Q G E P Q S K S P Q E S R
% of base peak
ion current
m/z
c
IP
Input
Igg
TPX2
siControl
SiCOASY
KDa
CBP
TPX2
COASY
Cyclin B1
GAPDH
250
100
75
50
50
37
d
Input
IP: V5
vector
TPX2-V5
CBP-HA
KDa
HA (CBP)
V5 (TPX2)
GAPDH
Ac-K (TPX2)
250
100
37
e
Cycloheximide
CBP Wt
CBP Mut
0 2 4 6 0 2 4 6 (hours)
KDa
V5 (TPX2)
β-tubulin
100
50
CBP WT
CBP Mut
TPX2 protein level
(normalized to β-tubulin)
Time (hours)
***
***
*
f
Input
IP: V5
CBP
V5-TPX2 WT
V5-TPX2 RRR
KDa
CBP (HA)
TPX2 (V5)
GAPDH
Relative
acetylation (%)
100
8.9
TPX2 (Ac-K)
250
100
37
g
Input
IP:V5
TPX2 WT-V5
TPX2 RRR-V5
MG132
Ub
V5 (TPX2)
GAPDH
KDa
100
37

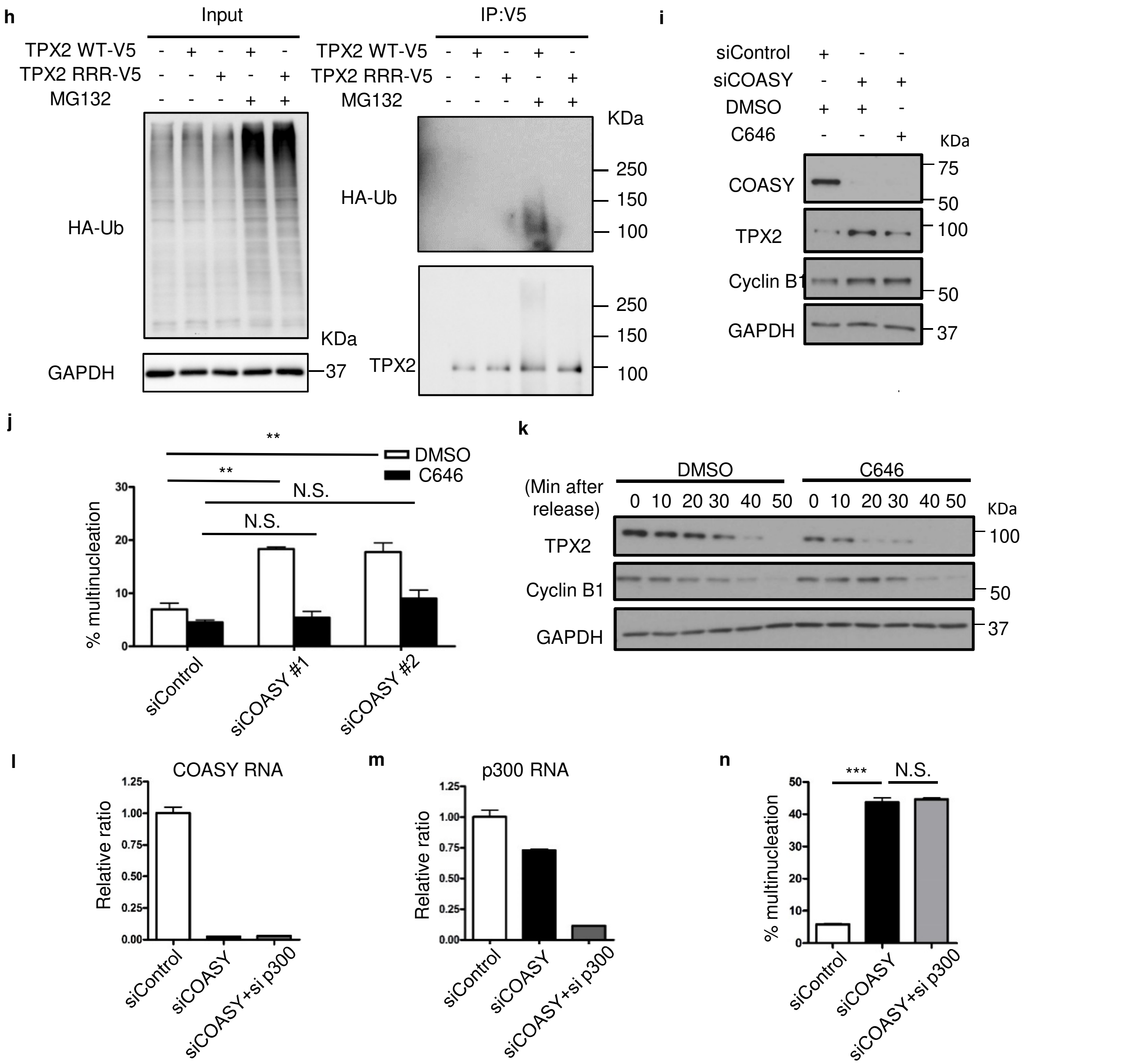

Supplementary Fig. 4 a, b MS/MS Spectra of two peptides in the region of K1762 of CBP, one with acetylation at K1744 terminating in K1762 (**a**), and one containing site-localized acetylation at K1762 (**b**).**c** COASY knockdown did not alter the CBP-TPX2 interaction in A549 cells. A549 cells enriched in early mitosis by thymidine-nocodazole block were transfected with control or COASY siRNA and probed with indicated antibodies. The TPX2 in the cell lysates were then immunoprecipitated and blotted for CBP antibody. **d** CBP acetylated TPX2 *in vivo* in MDA-MB-231 cells. HA-tagged CBP was cotransfected with V5 tagged TPX2 into HEK-293T cells. After 20 min of release from thymidine-nocodazole block, the V5 tagged TPX2 was then immunoprecipitated with V5 antibody and then probed with HA (CBP), acetylated lysine or V5 (TPX2). **e** CBP increased the half-life of TPX2 in A549 cells. Wild type or catalytic deficient mutant of CBP was cotransfected with V5 tagged TPX2 into unsynchronized A549 cells. After 24 hours, the protein synthesis of the transfected cells were halted with 25 mg/ml cycloheximide and collected at the indicated times for Western blots. The TPX2 protein level at indicated time points was quantified by Image J software and normalized to β-tubulin protein level in four independent experiments. **f** CBP increased TPX2 acetylation on three lysine residues on TPX2. Wild-type and triple mutant TPX2 (RRR, K75R, K476R and K582R) were co-transfected with CBP cDNA in HEK-293T cells. When the TPX2 protein was pulled down by V5 tag, triple mutant of TPX2 showed ~91% decrease in normalized acetylation than wild-type TPX2. Two-way ANOVA: $p<0.0001$. Bonferroni post hoc tests, *$p<0.05$, ***$p<0.001$. n=4 independent repeats. **g, h** Removal of the three potential acetylation residues on TPX2 decreased its ubiquitination. **g** HEK-293T cells transfected with wild-type or triple mutant TPX2 (RRR, K75R, K476R and K582R) were treated with MG132. TPX2 were then immunoprecipitated by V5 antibody and blotted for pan-ubiquitination antibody. **h** MDA-MB-231 cells co-transfected with HA tagged ubiquitin and wild-type or triple mutant TPX2 (RRR, K75R, K476R and K582R) were treated with MG132. After lysing cells in denaturing condition (1%SDS and boiling for 5 min),TPX2 were then immunoprecipitated by V5 antibody and blotted for ubiquitination by HA antibody. A broad range of ubiquitylated TPX2 species from 100-190 Kda. **i** C646 abolished the increased TPX2 caused by COASY knockdown. A549 cells with indicated COASY knockdown and/or C464 treatment were synchronized by thymidine-nocodazole block for Western blots. **j** C646 rescued the multinucleation, as determined by immunofluorescence, caused by COASY knockdown. **k** C646 decreased the induction of TPX2 protein during mitosis. A549 cells were synchronized by thymidine-nocodazole block with or without C646 (5 μM). After 16 hours of nocodazole arrest, the cells were released in fresh media with C646. The samples were then harvested every 10 min after the release for Western blots. **l, m** Validation of knockdown efficiency of COASY (**l**) and p300 (**m**) mRNA by real-time PCR. **n** p300 knockdown did not rescue the multinucleation caused by COASY knockdown. (**j, n**) **$p<0.01$, ***$p<0.001$, two-tailed Student's t-test, n=3 independent repeats. Bars show standard error of the mean.

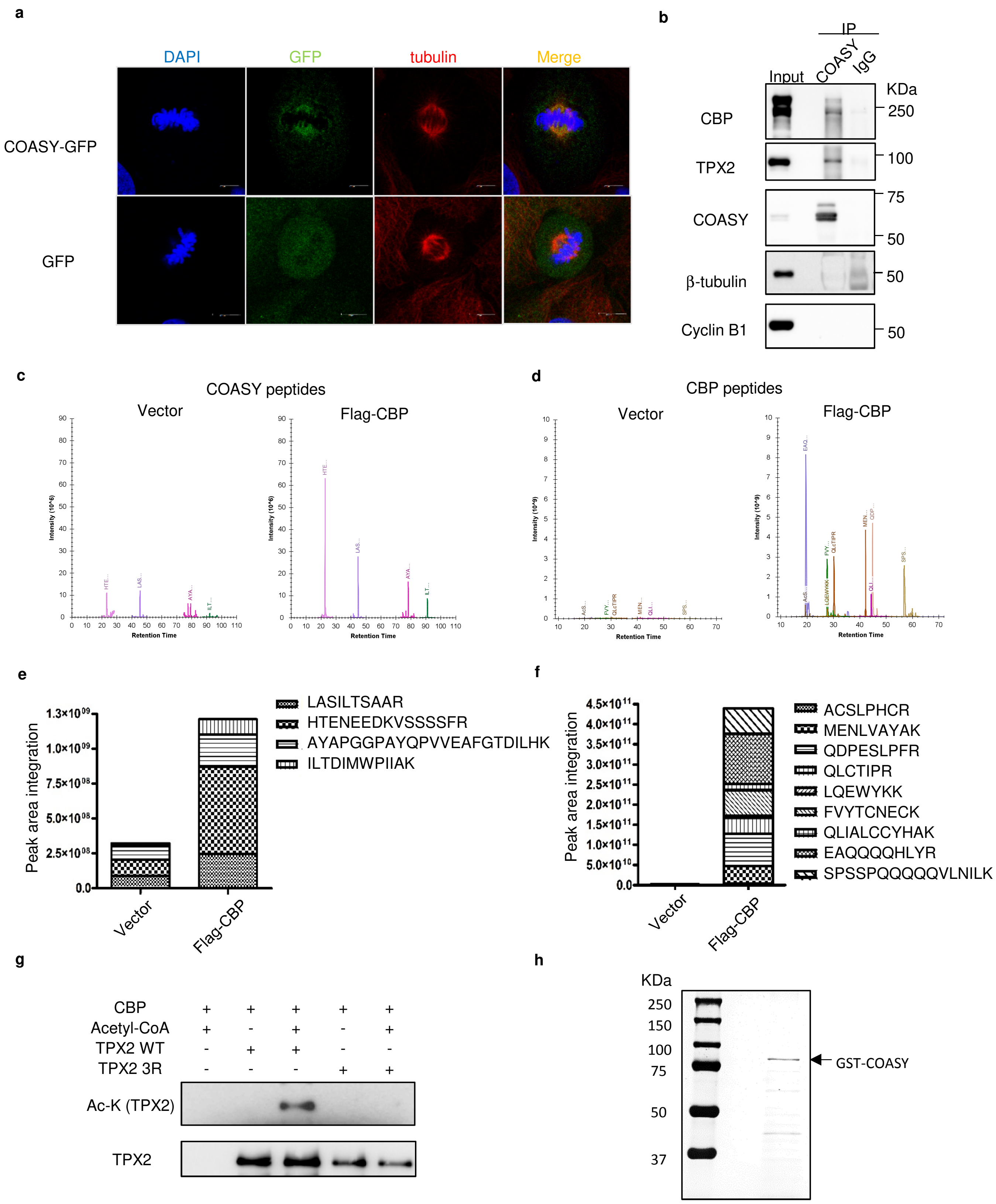

Supplementary Fig. 5 a GFP-tagged COASY, but not GFP alone, showed colocalization with tubulin during metaphase. **b** COASY formed a complex with CBP and TPX2. HEK-293T cells enriched in mitosis were harvested for co-immunoprecipitation of COASY antibody and probed with either CBP or TPX2 antibodies by Western blots. **c-f** Mass spectrometry confirmed enrichment of COASY peptides when Flag-CBP was pulled down from HEK-293T cells overexpressing Flag-CBP and COASY cDNA. **c, d** Extracted Ion Chromatograms for 4 COASY peptides (**c**) and 9 CBP (bait) peptides (**d**) between control and Flag-CBP expressing HEK-293T cells. **e, f** Bar Charts signifying the sum of the 4 COASY peptides (**e**) increased with pulling down of 9 Flag-CBP peptides (**f**). **g** CBP was unable to acetylate acetylation-deficient mutant (3R) of TPX2 *in vitro*. The recombinant catalytic domain of human CBP was incubated with wild type or acetylation-deficient mutant of TPX2 pulled down from HEK-293T cells in the presence of acetyl-CoA. Acetylation on TPX2 was determined by Western blots using pan-acetylated lysine antibody. **h** Bacterially expressed GST-COASY was purified and resolved by 10% SDS PAGE and silver staining. Human COASY cDNA was subcloned to an GST tagged vector (pGTvL1-SGC) for bacterial expression. Recombinant GST-COASY protein was induced in E. coli (BL21) and purified using glutathione agarose.

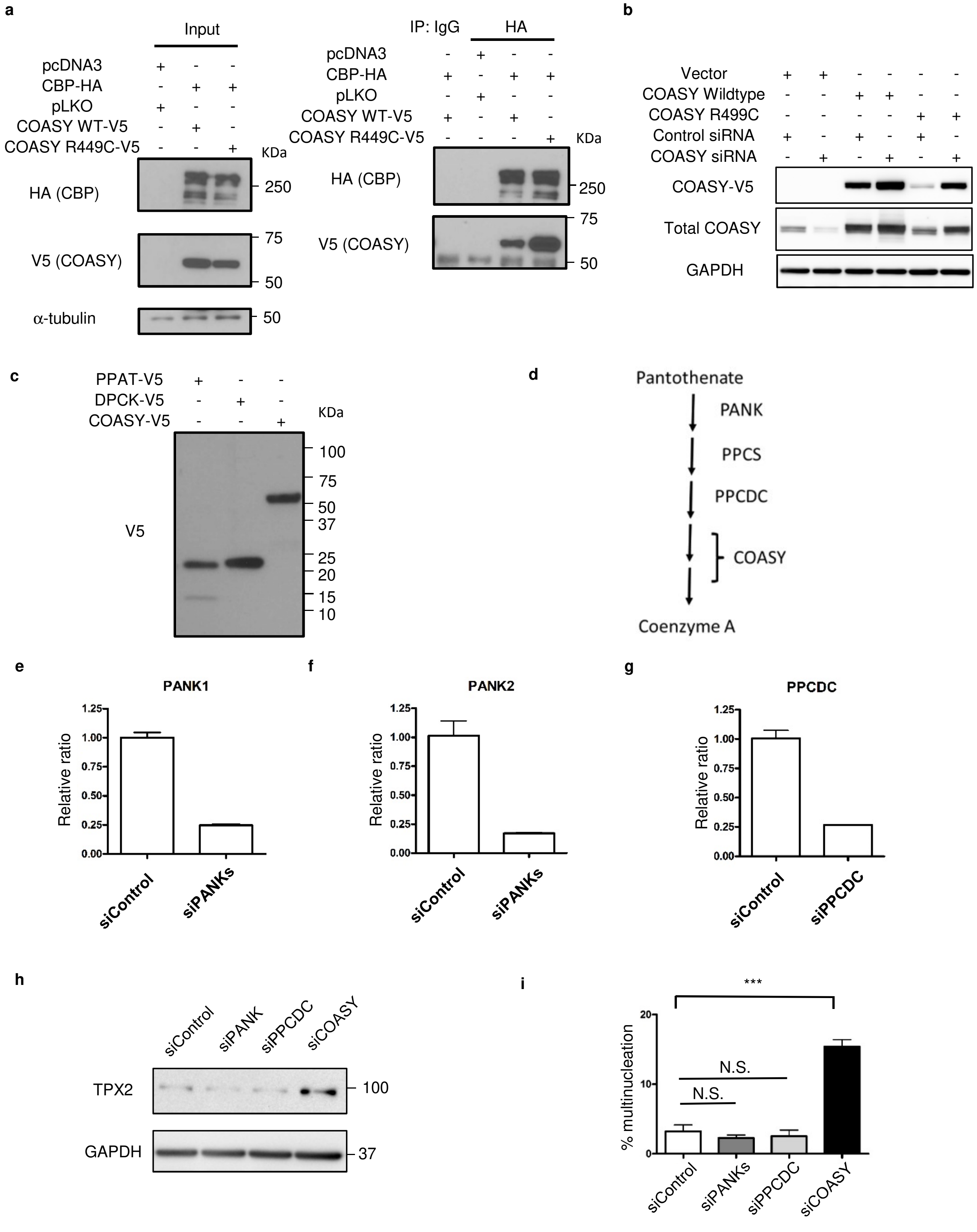

Supplementary Fig. 6 a Both wildtype or R499C mutant COASY protein showed strong interaction with CBP. CBP and COASY cDNAs were cotransfected to HEK-293T cells. The cells were then enriched in mitosis by 16 hours of nocodazole treatment. The samples were harvested for co-immunoprecipitation. **b** Western blot for validating the expression of indicated proteins in Fig 6B. **c** V5 tagged PPAT, DPCK and COASY cDNA were expressed with expected size in HEK-293T cells **d** A simplified chart of the enzymes involved in CoA biosynthesis that include pantothenate kinase (PANK), phosphopantothenoylcysteine synthetase (PPCS), Phosphopantothenoylcysteine Decarboxylase (PPCDC) and COASY A549 cells were treated pooled siRNAs that targeted COASY, PANKs and PPCDC for 72 hours. **e-g** Real-time PCR was performed to validate the efficient knockdown of PANK1 (**e**), PANK2 (**f**) and PPCDC (**g**) by respective siRNAs. PANK siRNA is a mixture of siRNA that targets both PANK1 and PANK2. **h, i** Inhibition of two upstream enzymes of CoA biosynthesis, PANKs and PPCDC, was unable to induce TPX2 upregulation (**h**) and multinucleation (**i**). N.S. not significant, ***p<0.001, two-tailed Student's t-test, n=3 independent repeats. Bars show standard error of the mean.

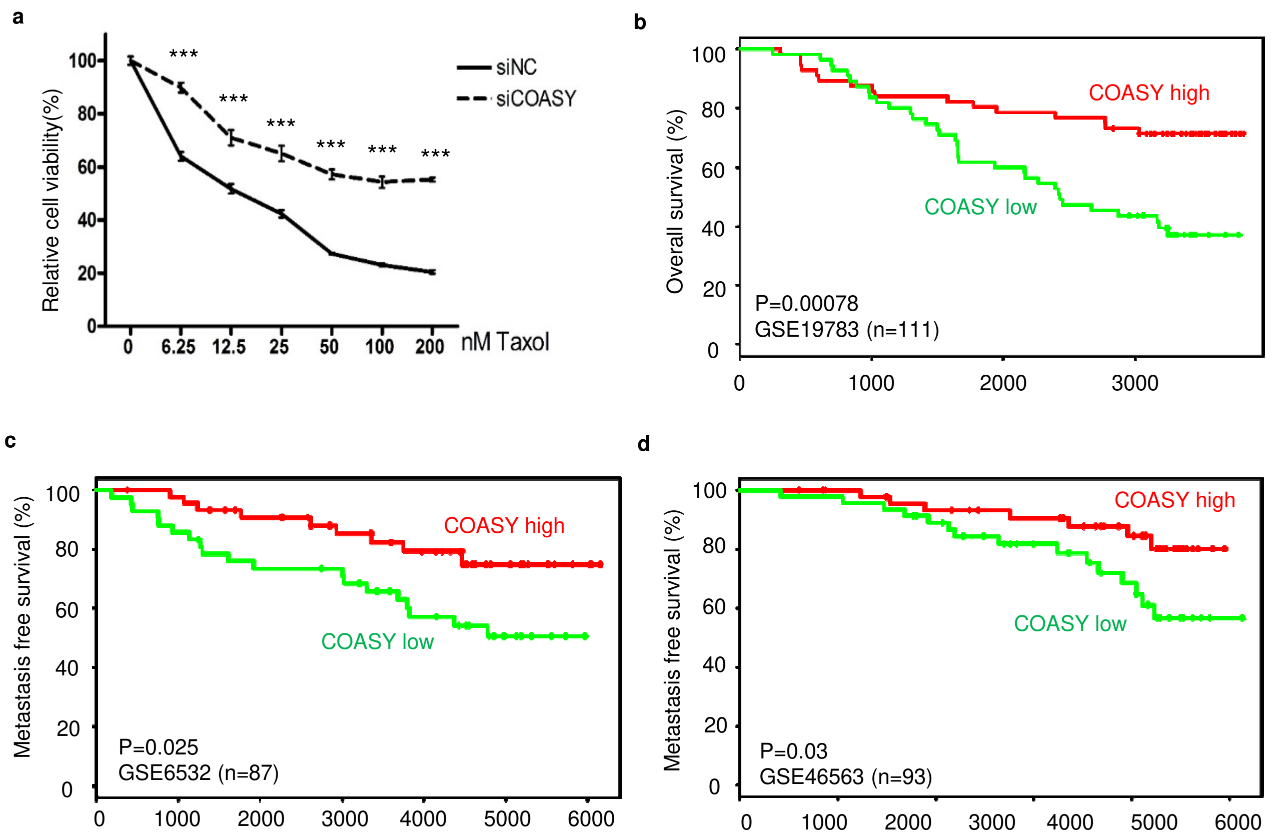

Supplementary Fig. 7 a COASY knockdown rendered MDA-MB-231 cells resistant to treatment of indicated levels of taxol. Relative cell viability was measured by CellTiter-Glo assay (Promega). Two-way ANOVA: $p<0.0001$. Bonferroni post hoc tests, ***$p<0.001$. n=3 independent repeats. **b-d** Low COASY expression in human tumors is correlated with decreased survival and metastasis. Breast cancer patients were bifurcated into high and low COASY expression groups by the median of COASY expression in the primary breast tumors and their clinical outcomes were compared by Kaplan-Meyer analysis in overall survival in the Enerly and Steinfeld dataset (GSE19783) (**b**), metastasis-free survival in the Loi dataset (GSE6532) (**c**), metastasis-free survival in in the Jonsdottir dataset (GSE46563) (**d**).

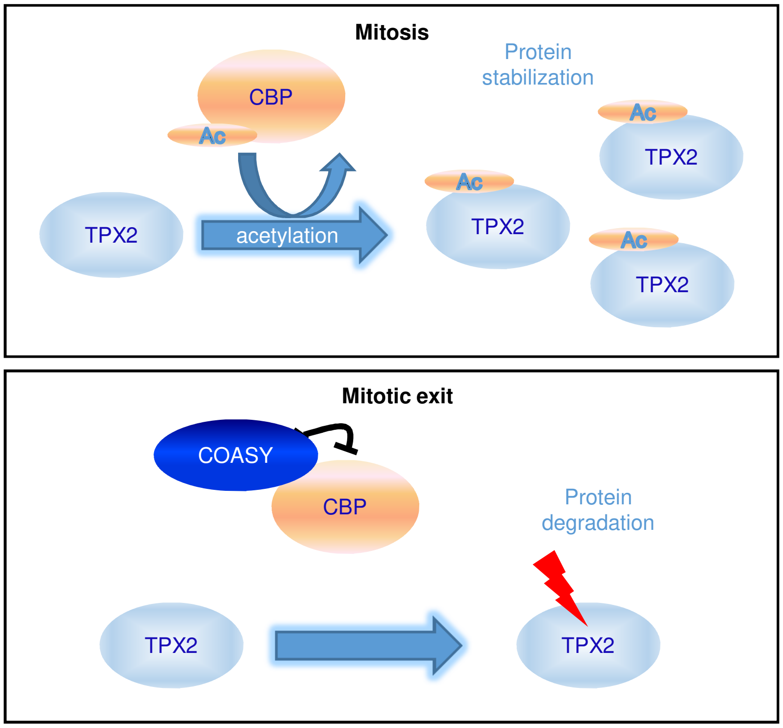

Supplementary Fig. 8 Schematic illustration of the COASY-CBP-TPX2 model.

Supplementary Fig. 9 Uncropped images of Western blot

Fig 1g

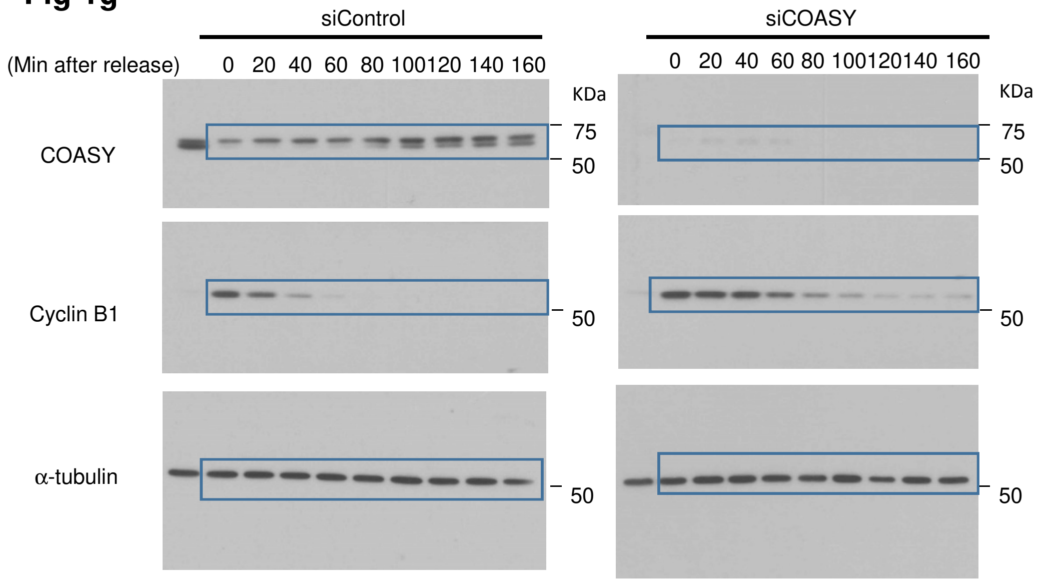

Fig 3b

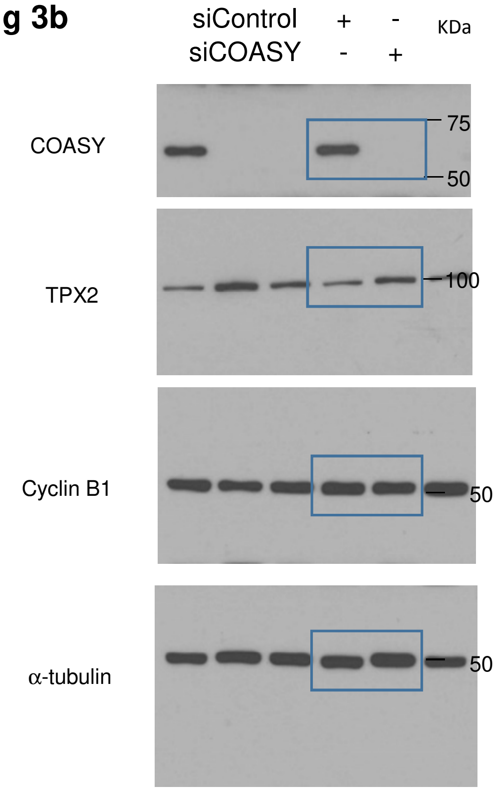

Fig 3c

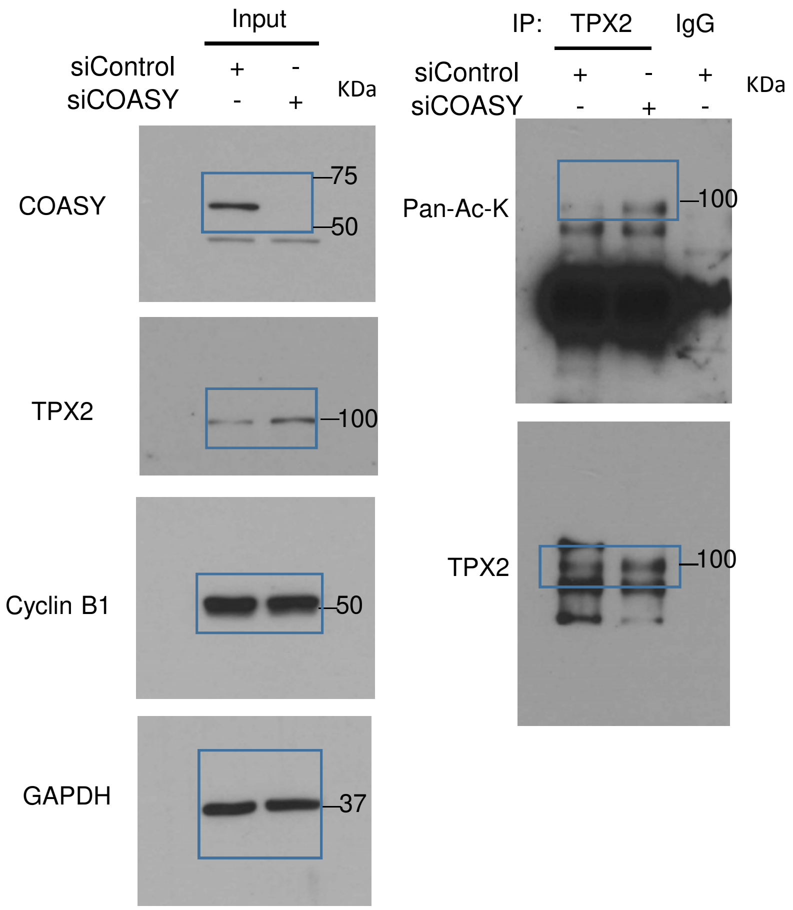

Input
siControl + -
siCOASY - +
KDa
COASY
75
50
TPX2
100
Cyclin B1
50
GAPDH
37
IP: TPX2 IgG
siControl + - +
siCOASY - + -
KDa
Pan-Ac-K
100
TPX2
100

Fig 3d

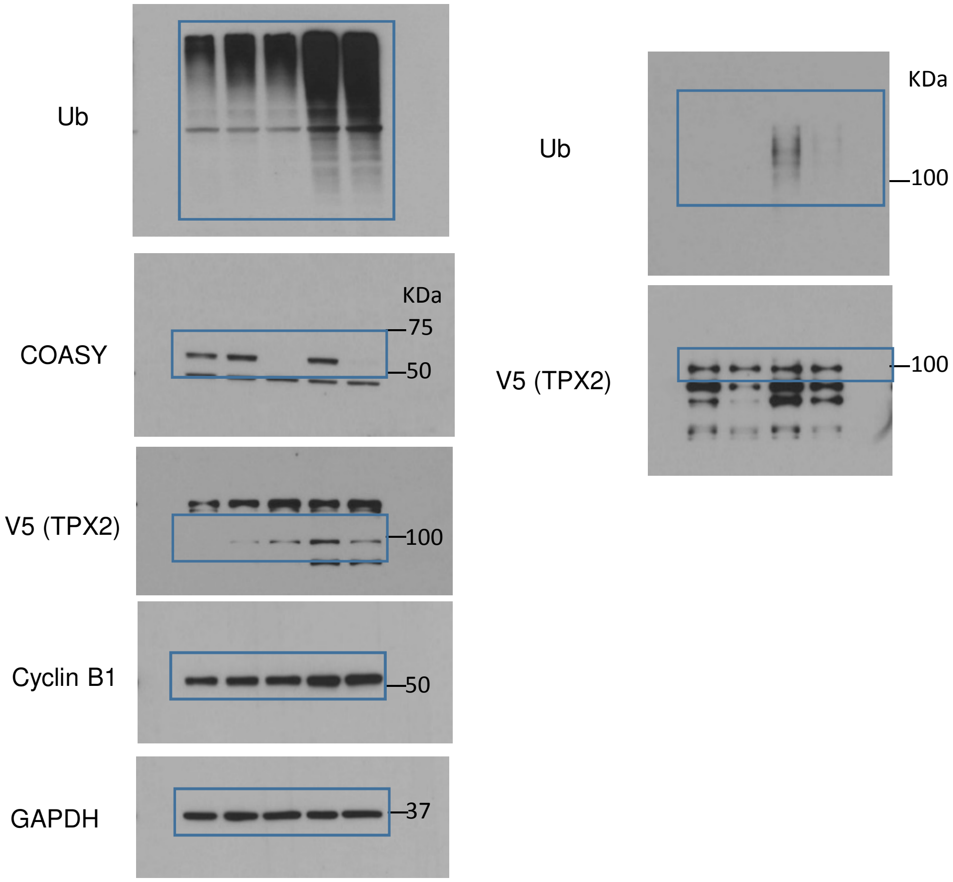

Ub
COASY
KDa
75
50
V5 (TPX2)
100
Cyclin B1
50
GAPDH
37
KDa
Ub
100
V5 (TPX2)
100

Fig 3e

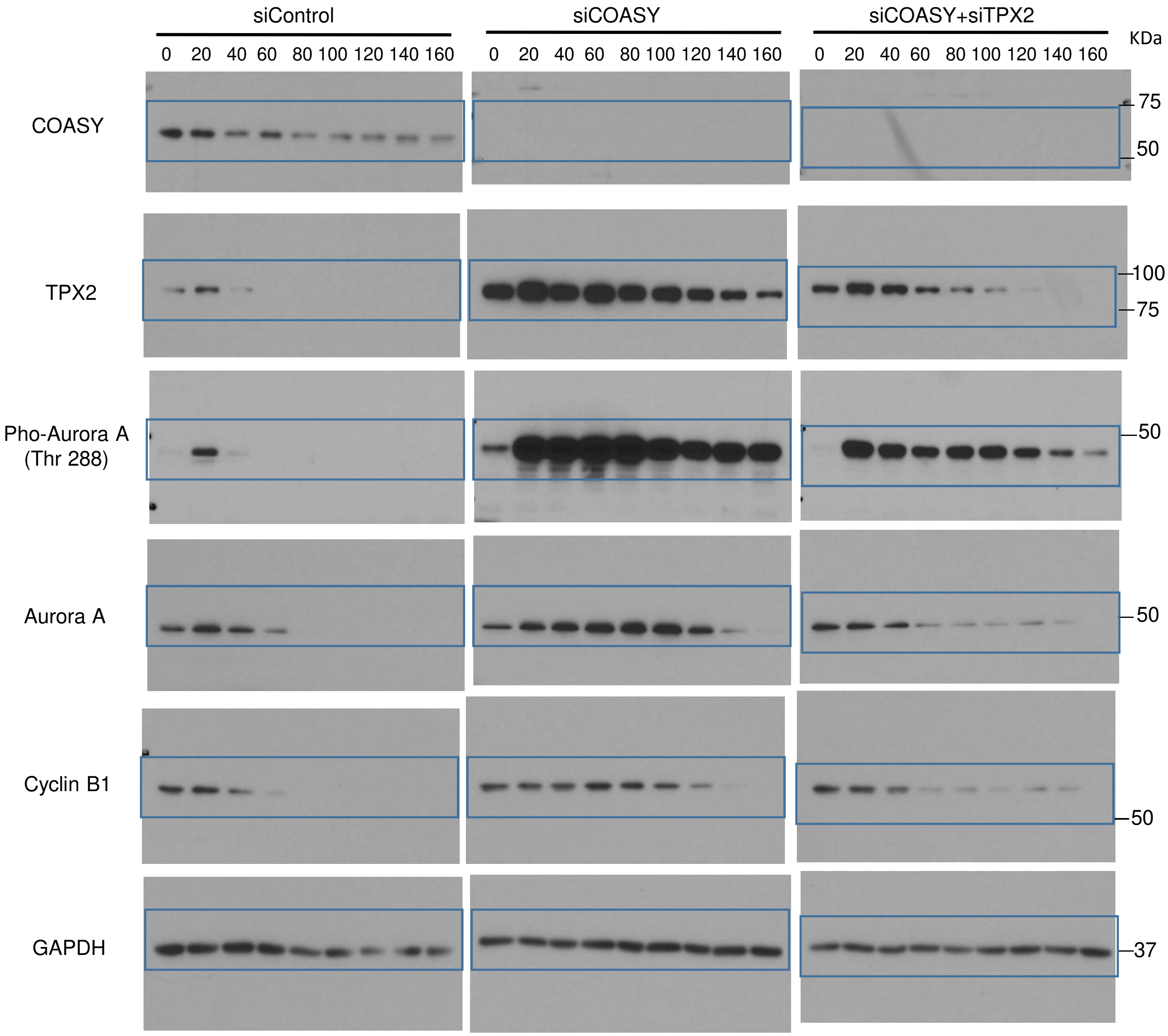

siControl
siCOASY
siCOASY+siTPX2
0 20 40 60 80 100 120 140 160
0 20 40 60 80 100 120 140 160
0 20 40 60 80 100 120 140 160
KDa
COASY
75
50
TPX2
100
75
Pho-Aurora A (Thr 288)
50
Aurora A
50
Cyclin B1
50
GAPDH
37

Fig 4b

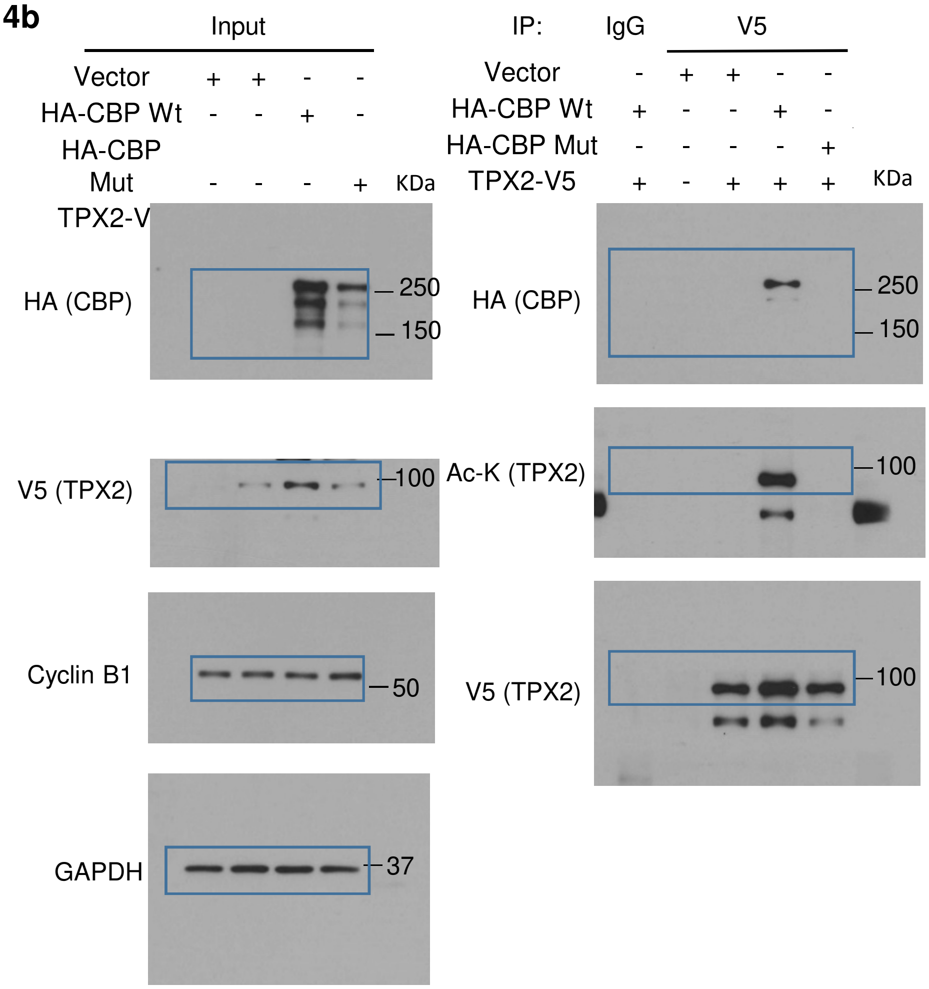

Input
Vector + + - -
HA-CBP Wt - - + -
HA-CBP Mut - - - +
TPX2-V
KDa
HA (CBP)
250
150
V5 (TPX2)
100
Cyclin B1
50
GAPDH
37
IP: IgG V5
Vector - + + - -
HA-CBP Wt + - - + -
HA-CBP Mut - - - - +
TPX2-V5 + - + + +
KDa
HA (CBP)
250
150
Ac-K (TPX2)
100
V5 (TPX2)
100

Fig 4c

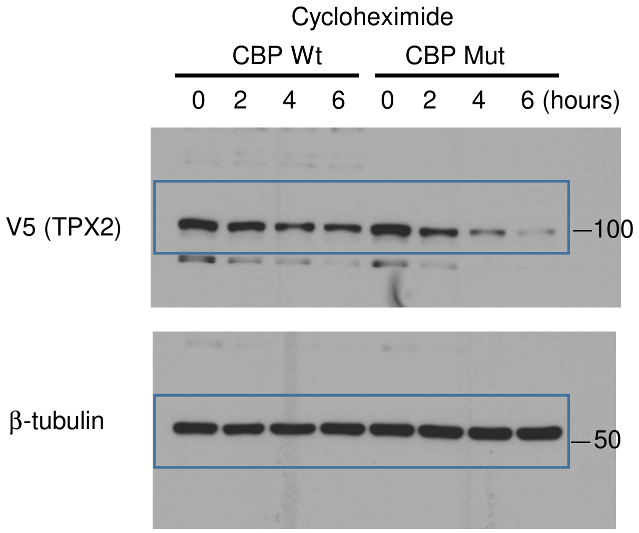

Cycloheximide
CBP Wt
CBP Mut
0 2 4 6 0 2 4 6 (hours)
V5 (TPX2)
100
β-tubulin
50

Fig 4d

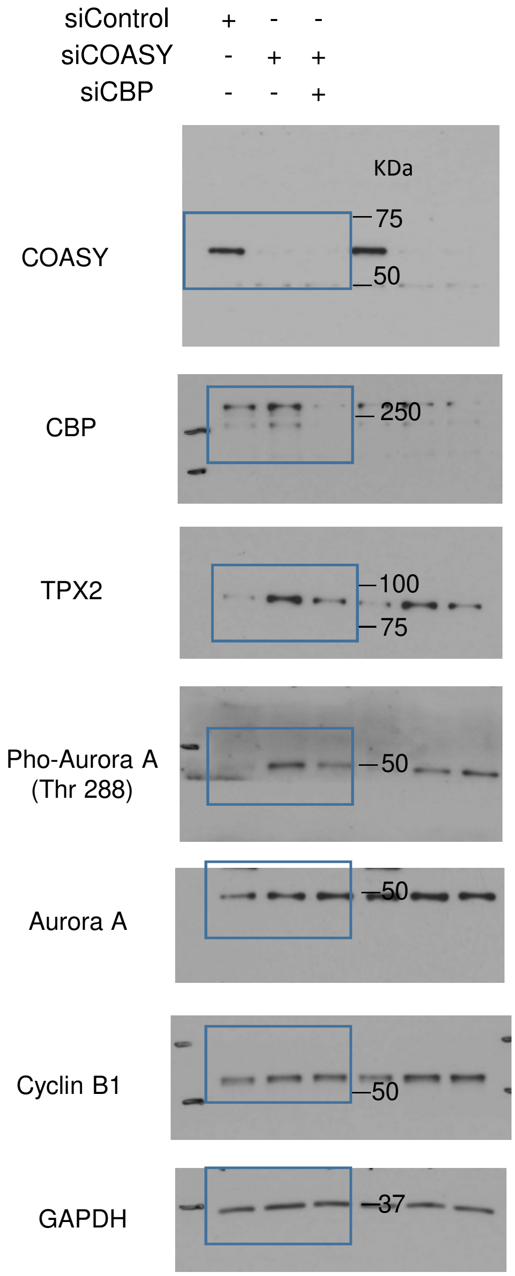
siControl + - -
siCOASY - + +
siCBP - - +
KDa
COASY
75
50
CBP
250
TPX2
100
75
Pho-Aurora A
(Thr 288)
50
Aurora A
50
Cyclin B1
50
GAPDH
37

Fig 4f

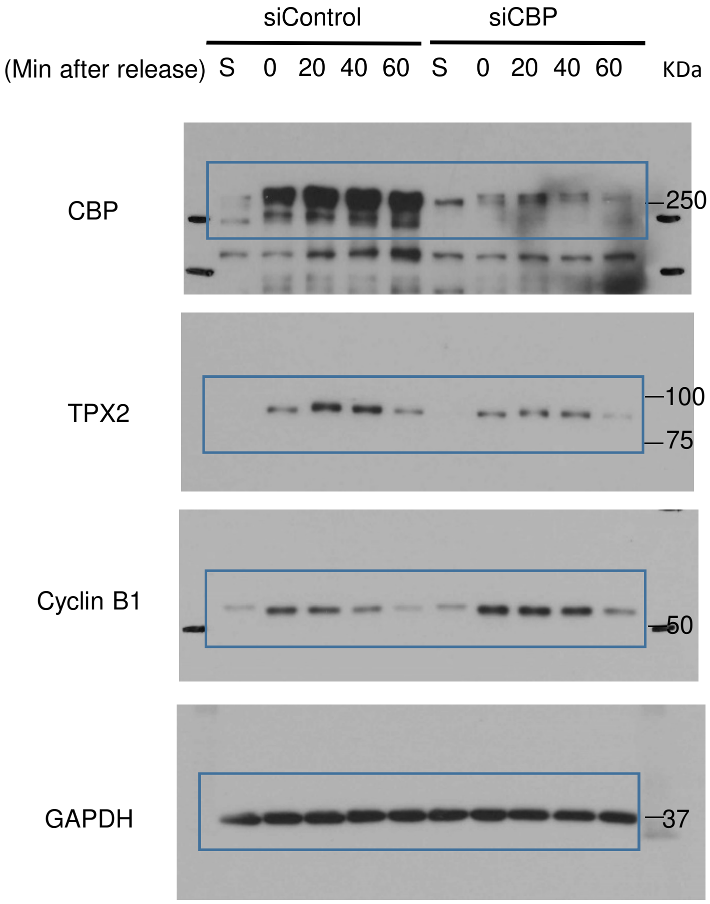
siControl
siCBP
(Min after release) S 0 20 40 60 S 0 20 40 60
KDa
CBP
250
TPX2
100
75
Cyclin B1
50
GAPDH
37

Fig 5c

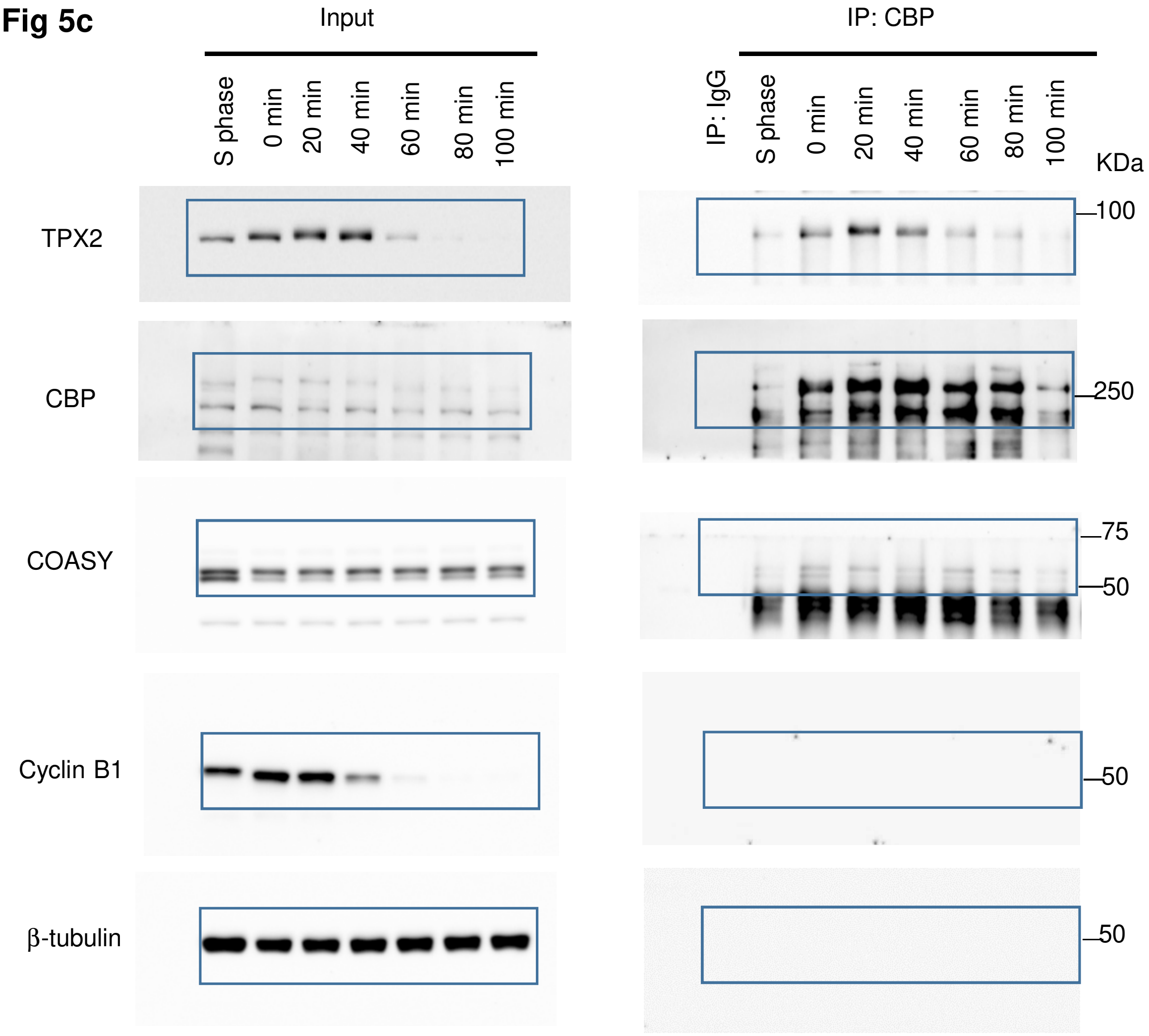

Input
IP: CBP
S phase
0 min
20 min
40 min
60 min
80 min
100 min
IP: IgG
KDa
TPX2
CBP
COASY
Cyclin B1
β-tubulin
100
250
75
50
50
50

Fig 5d

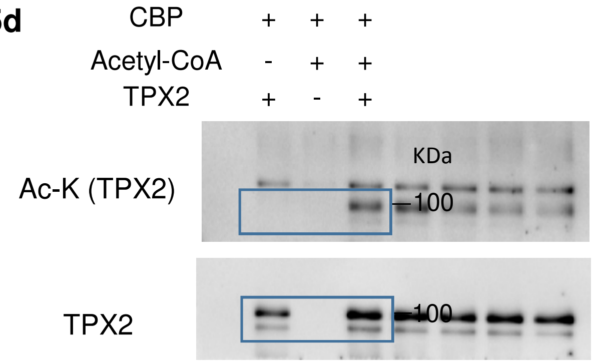

CBP + + +
Acetyl-CoA - + +
TPX2 + - +
Ac-K (TPX2)
TPX2
KDa
100
100

Fig 5e

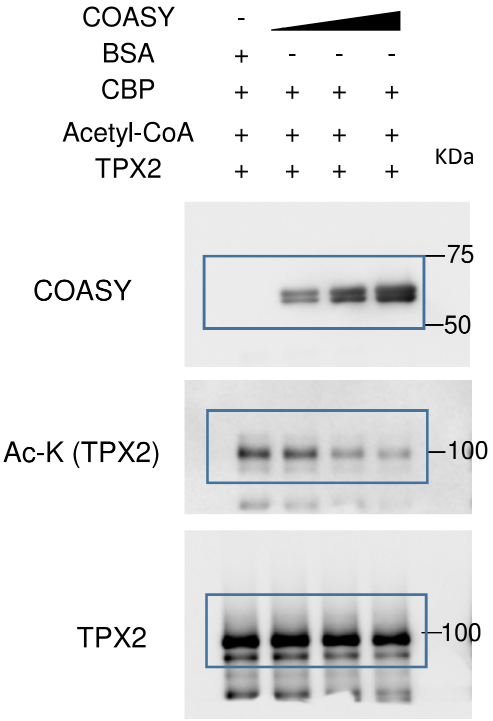
COASY -
BSA + - - -
CBP + + + +
Acetyl-CoA + + + +
TPX2 + + + +
KDa
COASY
75
50
Ac-K (TPX2)
100
TPX2
100

Fig 6d

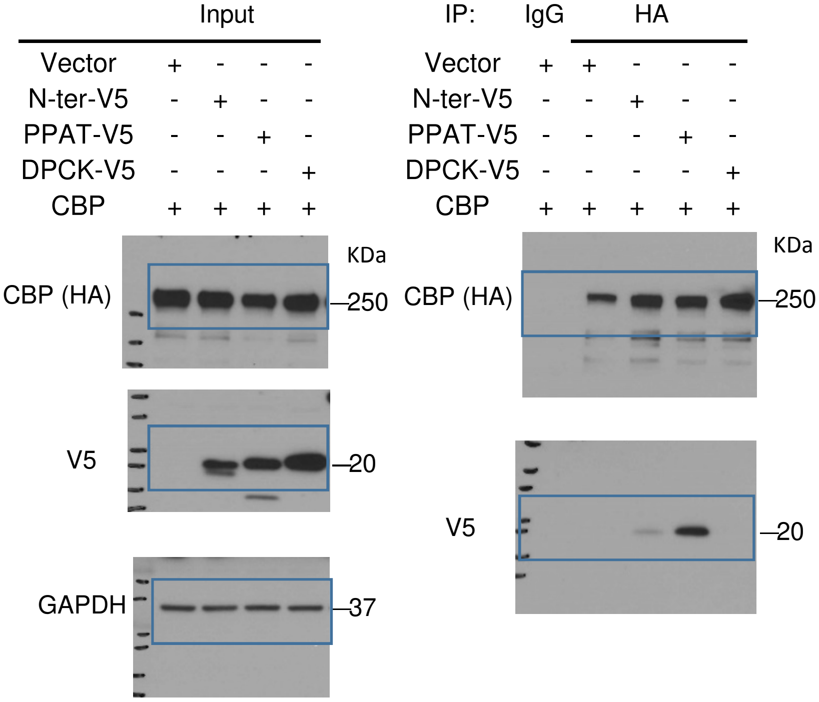
Input
IP: IgG HA
Vector + - - -
N-ter-V5 - + - -
PPAT-V5 - - + -
DPCK-V5 - - - +
CBP + + + +
Vector + + - - -
N-ter-V5 - - + - -
PPAT-V5 - - - + -
DPCK-V5 - - - - +
CBP + + + + +
KDa
CBP (HA)
250
V5
20
GAPDH
37

Fig 6e

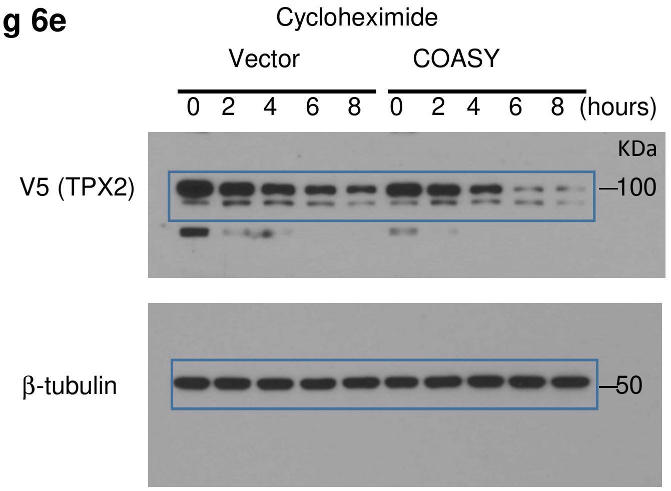

Fig 6f

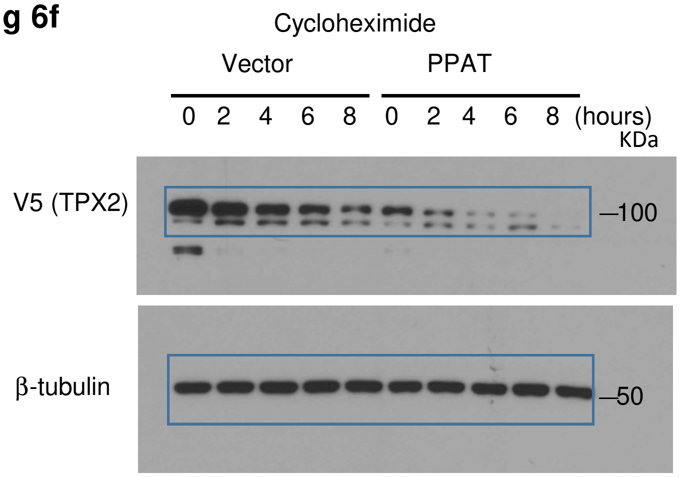

Fig 6g

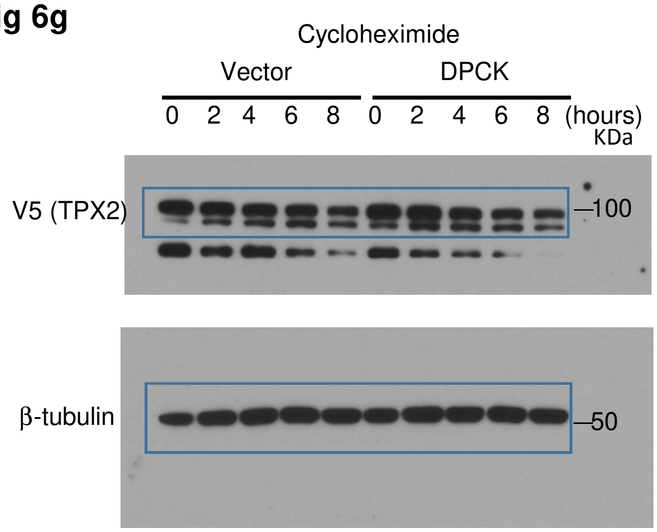

Supplemental Materials and Methods

Protein Isolation and Trypsin Digestion. The Duke Proteomics and Metabolomics Core Facility (DPMCF) received snap-frozen A549 cell pellets, three from WT and 3 from CoA Synthase (CoASY) knockdown via siRNA. Solubilization was performed with probe sonication in 1.7 mL 8M Urea buffered to pH 8 with 50 mM ammonium bicarbonate (AmBic). Samples were centrifuged at 15,000 rcf for 5 mins. Protein yield was determined using a mini-Bradford Assay (Bio-Rad), for each sample in duplicate, and these results are shown in Supplementary Table 1. Approximately 7 mg protein for each sample was concentration-normalized to equivalent concentration and volume, with dilution to 1.8 M Urea/ 50 mM AmBic using 50 mM AmBic. Supplementary Table 1 records Sample ID from the Proteomics Core LIMS, sample name (as delivered), and initial protein concentration after solubilization. The target protein quantity for the immunoprecipitation was 5 mg, so all samples yielded sufficient protein to proceed.

Proteomics ID (Raw Data)	Sample Name	[c] in 8 M urea (mg/mL)	Analysis Order
ID13410	NC 1	6.24	1,7
ID13411	NC 2	6.39	3,9
ID13412	NC 3	6.95	5,11
ID13413	COASY 1	5.42	2,8
ID13414	COASY 2	4.33	4,10
ID13415	COASY 3	5.31	6,12

Supplementary Table 1. Protein Yield from Lysis of A549 Cell Pellets in 8 M Urea buffered with 50 mM ammonium Bicarbonate.

Each sample was divided into four Eppendorf tubes each containing 1.75 mg protein in 404 uL (normalized using 8M Urea in 50 mM AmBic). Then each tube was diluted to 1796 uL with AmBic to get to 1.8M urea final for the purposes of trypsin digestion. Prior to digestion, each tube was spiked with 7.5 ng (7.5×10^{-9} g) acetylated bovine serum albumin (Ac-BSA), a standard generated by Duke Proteomics Core by reacting BSA with NHS-acetate (Pierce). Cysteine residues were reduced with 10 mM dithiothreitol for 45 min at 32°C, and alkylated at 20 mM iodoacetamide for 30 min at RT in the dark. 67 ug TPCK trypsin was added to each eppendorf tube (25:1 substrate:enzyme ratio), and digestion was allowed to proceed at 32°C overnight. Samples were then acidified to 0.5% v/v TFA, and desalted using 500 mg SEP-PAK C18 cartridges (Waters), per manufacturer's protocol. 2.25 mL peptide-containing eluate (41.7/58.3/0.1 v/v/v MeCN/H_2O/TFA) was diluted with 3 mL water, then frozen at -80C and lyophilized overnight to yield peptide ready for immunoprecipitation.

Kac Peptide Immunoprecipitation. 7 mg digested peptide (with Ac-BSA internal standard) for each sample was resuspended in 1.4 mL IAP Buffer (Cell Signaling Technology) using vortex and brief bath sonication, and transferred to an aliquot of Acetyl-K PTMScan enrichment beads (Cell Signaling Technology). IP was performed for two hours at 4C using end-over-end mixing. After spinning to settle the beads and removing the supernatant, Ac-K enrichment beads were washed with a single aliquot of 1% NP40 in IAP buffer, one aliquot of IAP buffer, and three times with water. Elution was performed with two 50 ul aliquots of 0.15% TFA in water, for approximately 10 minutes at room temperature. Eluates were combined and taken through a C18 STAGE tip desalting cleanup, and resulting peptides were dried via speedvac. Samples were finally resuspended in 12 ul of 1/2/97 v/v/v/TFA/MeCN/H2O containing 10 fmol/uL ADH1_YEAST MassPrep Standard (Waters) and transferred to autosampler vials.

Quantitative analysis Kac proteome. Quantitative LC/MS/MS was performed in duplicate for each sample (4 ul per injection), using a nanoAcquity UPLC system (Waters Corp) coupled to a Q-Exactive Plus high resolution accurate mass tandem mass spectrometer (Thermo Scientific) via a nanoelectrospray ionization source. Briefly the sample was first trapped on a Symmetry C18 20mm × 180 um trapping column (5 μl/min at 99.9/0.1 v/v

water/acetonitrile), after which the analytical separation was performed using a 1.7 um Acquity HSS T3 C18 75 um × 250 mm column (Waters Corp.) using a 90-min gradient of 5 to 40% acetonitrile with 0.1% formic acid at a flow rate of 400 nanoliters/minute (nL/min) with a column temperature of 55C. Data collection on the Q-Exactive Plus was performed with MS1 (precursor) analysis at 70,000 resolution and AGC target of 1e6 ions. Tandem mass spectra (MS/MS) were collected in a data-dependent manner on the top 10 most abundant precursor ions per scan at a resolution of 17,500 using HCD collision energy of 27, an AGC target 5e4 ions, and a max MS2 accumulation time of 60 msec. Dynamic exclusion was enabled with a 20 second exclusion window.

Sample order of data collection was interwoven between conditions in order to minimize temporal bias, and run order is shown in Supplementary Table 1. Duplicate analyses for each sample were run separately in order to accurately assess technical versus biological variance during the analysis time. Following the 12 analyses, data was imported into Rosetta Elucidator v3.3 (Rosetta Biosoftware, Inc), and all LC-MS/MS runs were aligned based on the accurate mass and retention time of detected ions ("features") using PeakTeller algorithm (Elucidator). Default settings in the software were used with the exception of the following: minimum ion intensity threshold was set to 10,000, peak intensity measurement was made based on peak area, the minimum peak time score was set to 0.5, and only MS1 features with at least one MS/MS event throughout the dataset were analyzed. The relative peptide abundance was calculated based on area-under-the-curve (AUC) of aligned features across all runs. The overall dataset had 37,646 quantified isotope (peptide) groups, and 272,641 MS/MS spectra were acquired for peptide sequencing by database searching. This MS/MS data was searched against the Uniprot/reviewed sequence database (www.uniprot.org) with *homo sapiens* taxonomy. The database also contained a reversed-sequence "decoy" database for false positive rate determination as well as several proteins which are either common lab contaminants or surrogate standards (ADH1_YEAST, ALBU_BOVIN, PYGM_RABIT, CASA1_BOVIN, and ENO1_YEAST). Database searching was performed in an automated fashion from the Elucidator software package using Mascot search engine (v2.4), assigning precursor ion tolerance of 5 ppm and product ion tolerance of 0.02 Da. Searching allowed variable modification of N and Q (deamidation, +1 Da), M (oxidation, +16 Da), and K (acetylation, +42 Da). After aggregating all search results, annotation (assignment of peptide sequence to the quantitative MS signal) was performed for peptides whose Mascot ion score was greater than 20, with precursor mass accuracy deviation less than 4 ppm from theoretical. This resulted in a total of 8084 peptides mapping to 2683 proteins, including 1074 peptides containing at least one acetylated K residue, at 1% peptide false discovery rate. For quantitative processing, the data was first curated to contain only high quality peptides with appropriate chromatographic peak shape (Supplementary Data 1). These Kac peptides belong to a total of 504 acetylated proteins. For differential quantitative analysis, only the acetyl-K peptides were selected (n=1074) and robust mean normalization (top and bottom 10% signals excluded) was performed in order to reduce quantitative bias; the normalized values are expressed in Supplementary Data 2, and these values were used for all subsequent statistical processing. The raw data and spectrum files for the discovery experiment have been uploaded to the MassIVE proteomics archive and can be downloaded at ftp://massive.ucsd.edu/MSV000081937.

Data QC and Outlier Screening. To assess system variability, %CV of peptide-level data was used. First, the non-normalized expression data (Supplementary Data 1) was used to assess global variability for yeast alcohol dehydrogenase (ADH) which was spiked at the same quantity into each of the 6 samples immediately before analysis (a measure of instrument (LC-MS/MS) system reproducibility); this average CV was 24% (n=20 peptides). Second, Ac-BSA peptides spiked in immediately after lysis were measured (a measure of total analytical variance including digestion, IP, SPE and LC-MS/MS); this average was 37% (n=92). Finally, the average %CV for all peptides in the dataset was used to assess total biological variance; this average was 47% (n=8084). After robust mean normalization of the Kac peptides only, the average biological variation within each group was found to be 30%, which is quite acceptable for differential expression analysis.

Additionally, the fraction of the total abundance within the LC-MS/MS analysis which was due to peptides of each type (acetylated, acetylated from BSA, and non-acetylated) was examined prior to intensity scaling (Supplementary Data 2) in order to assess the overall enrichment specificity and reproducibility. The enrichment for acetylated peptides by antibody IP had an overall specificity of 20±3%. The fraction of the IP which were Kac peptides from the exogenously-spiked acetylated BSA (30 ng total) was 1.7±0.2%, a quantity low enough to not interfere significantly with the IP of native peptides, but high enough to give good signal for the Kac-BSA standard. Finally, the fractional abundance of Kac peptides between the WT versus CoASY condition for the unscaled data can be used to assess whether there is a global shift in acetylation levels, since higher levels of global acetylation would be expected to yield higher enrichment specificity. Analyzing the Kac peptide fraction, there was a slight

trend towards higher specificity in the CoASY sample set (t-test p-value 0.02, effect size 1.3), suggesting that CoASY removal may be shifting the balance to slightly higher global protein acetylation levels. Analysis of the Kac-BSA peptides in the same manner showed no significant change (t-test p-value 0.89), suggesting any global acetylation level changes did not affect the Kac peptide IP yield.

To screen for outliers, each individual sample was plotted on a PCA plot for the top three principal components based on z-scored transformed peptide intensity, using the 1074 acetylated peptides in the. The PCA including all samples did not indicate any analytical outliers in relation to the other samples, and replicate analyses of the same sample were grouped together as expected. The most significant variance component (PC1) showed a very nice separation between WT and CoASY knockdown, suggesting that the challenge of interest is the most significant biological variable in the study.

Initial Statistical Processing. As described above, the data was intensity scaled at the peptide-level to the robust mean (exluding the highest and lowest 10% of the signals) across all injections, resulting in a final quantitative dataset for 1087 Kac peptides (Supplementary Data 2). Supplementary Data 2 also contains a first-pass statistical analysis of WT versus CoASY knockdown at the peptide level. The fold-change was calculated as the simple ratio of the average expression of the WT group to the CoASY group, where positive values reflect relative upregulation in WT and negative values reflect upregulation in CoASY. Next, p-values for significance of the change were calculated using a Two-tail Students T-test, after log2 scaling of the expression data (Supplementary Data 2). P-values have been corrected for multiple hypothesis testing using Benjamini-Hochberg FDR. Appropriate fold-change and p-value cutoffs were established with the assistance of a volcano plot, as shown in Supplemental Figure 10. This plot shows –log(10) of the p-value plotted against the fold-change; all native peptides in the sample are plotted in black and peptides to the exogenously spiked ac-BSA are shown in red. Peptides to ac-BSA are known to be in biologically identical quantities, so p-value and fold-change cutoffs were established to give a low quantitative false discovery rate (qFDR) of 1%. Empirically, we observed that at a fold-change cutoff of 1.75 and a p-value threshold of 0.001, the qFDR value was 0.8% (1/118); these thresholds are shown as dashed lines in Supplemental Figure 10. With these thresholds, 117 endogenous acetylated peptides from 90 proteins are putative differentially expressed candidates.

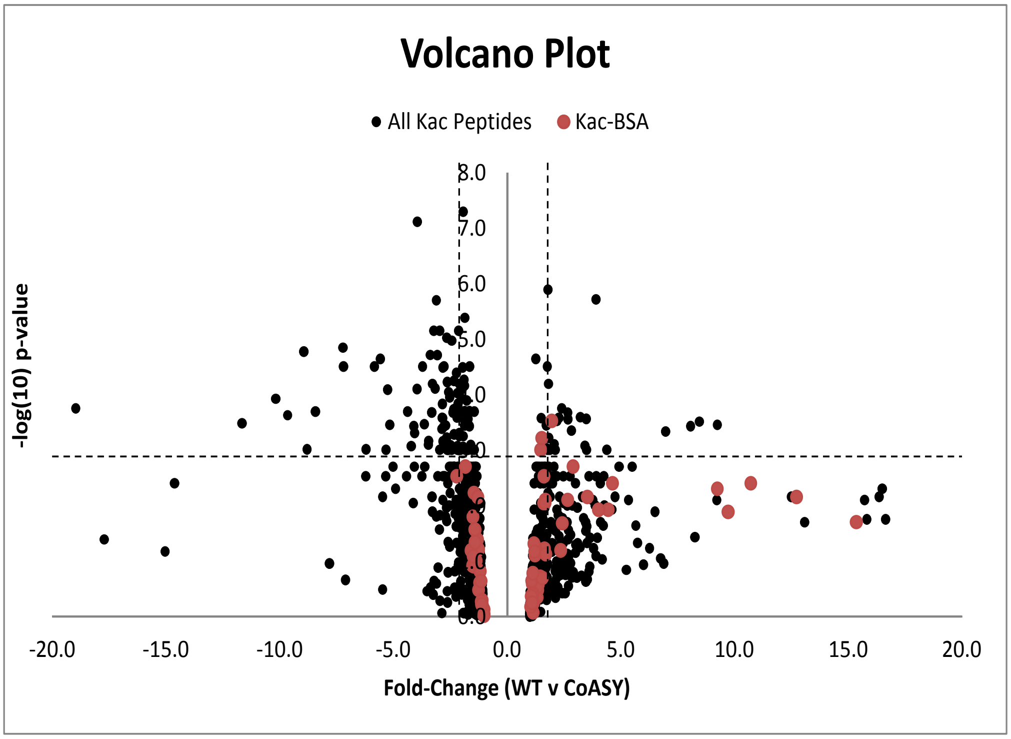

Supplemental Figure 10. Volcano Plot. Expression data for acetylated peptides (Supplementary Data 2) was used to calculate a fold-change (WT v CoASY), and t-test p-value (as described in the text). The fold-change and –log10 p-value was used to generate a volcano plot. Shown in black are the acetylated peptides from native proteins, and shown in red are acetylated peptides to Ac-BSA (spiked at equal levels into all samples.) The Ac-BSA measurements were used to define regions of true biological differential expression. Using this plot, a fold-change of 1.75 and p-value of 0.001 was selected

Global Expression Profiles. To assess the global expression profiles, a two-dimensional agglomerative cluster analysis of acetylated peptide expression levels (Supplementary Data 2) was performed using z-score transformed data. The 2D Cluster is shown in Supplementary Figure 11. Based on this clustering, it is clear that there is a significant and consistent difference in the global acetylation pattern between WT and CoASY siRNA cells, because even without statistical filtering, the biological replicates of each cluster together

appropriately. In the cluster we do not notice any significant outliers. In this analysis, similar to the observation based on peptide enrichment specificity levels, we do see a higher number of peptides being upregulated in the CoASY siRNA samples than in that of the WT, which is suggestive of a slight global shift towards higher levels of acetylation in the CoASY siRNA sample set.

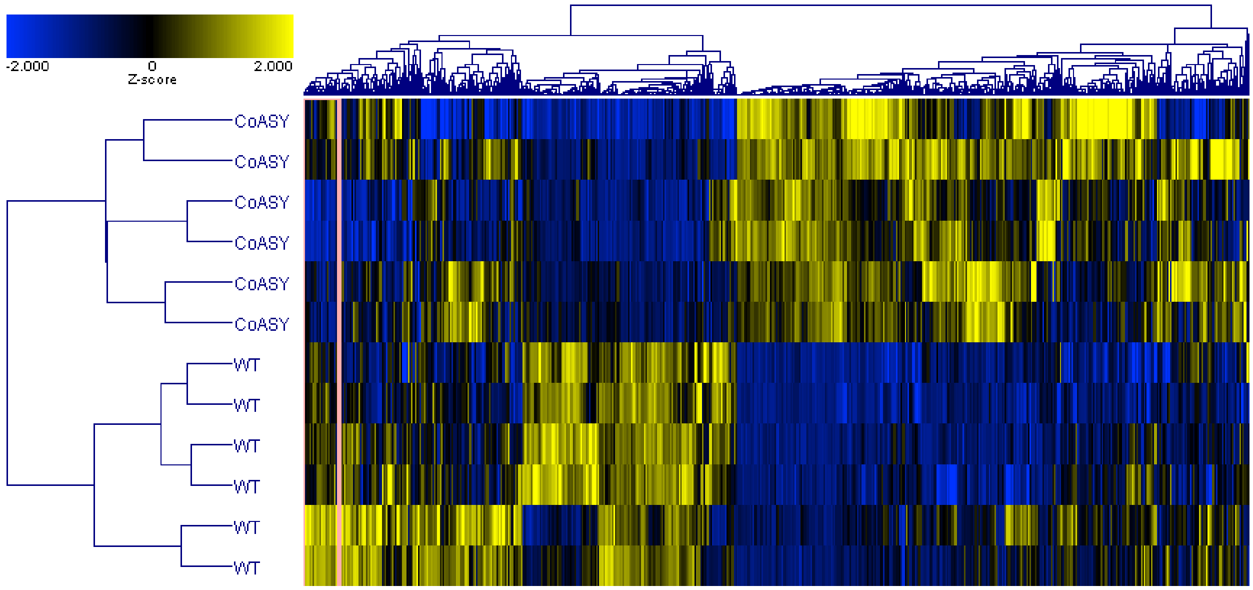

Supplementary Figure 11. 2D agglomerative clustering across all sample injections and acetylated peptides (n=1074). Z-score normalization of the values in Supplementary Data 2 was used prior to hierarchical clustering.
