# Supplementary material for: CoA synthase regulates mitotic fidelity via CBP-mediated acetylation
Source: Nat Commun. 2018 Mar 12;9:1039. doi: 10.1038/s41467-018-03422-6 (PMC5847545; doi:10.1038/s41467-018-03422-6)
Supplement: Supplementary file 3 — Description of Additional Supplementary Files [file 41467_2018_3422_MOESM3_ESM.pdf]

Description of Additional Supplementary Files

File Name: Supplementary Movie 1
Description: : Live cell time-lapse imaging showed that COASY knockdown extended mitosis and induced cytokinesis failure. A549 cells expressing histone 2B (H2B)-mCherry (nucleus marker) were transfected with control siRNA for 24 hours before live cell imaging.

File Name: Supplementary Movie 2
Description: A549 cells expressing histone 2B mCherry were transfected with COASY siRNA for 24 hours before live cell imaging.

File Name: Supplementary Data 1
Description: Peptide sequences and intensity of total acetylated peptides identified by acetylome analysis in synchronized A549 cells treated with control or COASY siRNAs.

File Name: Supplementary Data 2
Description: Peptide sequences and intensity of the acetylated peptides with statistical significance when treated with control or COASY siRNAs in synchronized A549 cells.
